# Highly-efficient VCSEL breaking the limit

**DOI:** 10.1038/s41377-024-01455-9

**Published:** 2024-05-28

**Authors:** Dieter Bimberg, Fumio Koyama, Kenichi Iga

**Affiliations:** 1 TU Berlin, Hardenbergstr. 36, 10623 Berlin, Germany; 2 Bimberg Chinese-German Center for Green Photonics, Changchun Institute of Optics, Fine Mechanics and Physics, Chinese Academy of Sciences, No. 3888 Dong Nan Hu Road, Changchun, 130033 China; 3 Tokyo Institute of Technology, Nagatsuta 4259, Midori-ku, Yokohama, 226-8501 Japan

**Keywords:** Optoelectronic devices and components, Photonic devices

*Light: Science & Applications* (2024)

10.1038/s41377-024-01403-7

VCSELs have become indispensable in the realms of datacenter networks and three-dimensional sensing thanks to their high-energy efficiencies, low-cost and the scalability of their two-dimensional arrays. VCSELs boast high energy-efficiency, exceeding 50% at low bias currents, superiority over edge-emitting lasers, particularly for optical interconnects within datacenter networks. However, there has been no breakthroughs in the efficiency of VCSELs for over a decade. With the swift evolution of AI clusters in datacenters and 3D-sensing for autonomous driving, where VCSELs are predominantly favored, the demand for ultra-high-efficiency VCSELs has become paramount. A team of researchers from Sichuan University and Suzhou Everbright Photonics Co., Ltd., have found a novel approach based on multiple-tunnel junctions within the active region. They demonstrated a record high-efficiency of 74% with prospects reaching as high as 88%. This breakthrough has the potential to reshape the 3D-sensing and datacenter ecosystems, providing a low-cost, energy-efficient, and reliable forthcoming generation of green photonics. The structural simplicity combined with its exceptional performances opens up a new era of VCSEL photonics with boundless opportunities for further research and innovation.

